# Urinary Lactate Dehydrogenase Activity and Its Isozyme Patterns in Kawasaki Disease

**DOI:** 10.1155/2017/4162597

**Published:** 2017-02-28

**Authors:** Yoichi Kawamura, Seiichiro Takeshita, Takashi Kanai, Mari Takizawa, Yusuke Yoshida, Yuki Tsujita, Shigeaki Nonoyama

**Affiliations:** ^1^Department of Pediatrics, National Defense Medical College, Tokorozawa, Saitama, Japan; ^2^Division of Nursing, National Defense Medical College, Tokorozawa, Saitama, Japan; ^3^Department of Pediatrics, Japan Self-Defense Forces Central Hospital, Setagaya, Tokyo, Japan

## Abstract

Abnormal urinary findings, such as sterile pyuria, proteinuria, and microscopic hematuria, are often seen in the acute phase of Kawasaki disease (KD). We investigated the potential significance of urinary lactate dehydrogenase (U-LDH) activity and its isozyme patterns in KD. Total U-LDH activity and its isozymes (U-LDH1-5) levels were compared among 120 patients with KD, 18 patients with viral infection (VI), and 43 patients with upper urinary tract infection (UTI) and additionally compared between intravenous immunoglobulin (IVIG) responders (*n* = 89) and nonresponders (*n* = 31) with KD. Total U-LDH activity was higher in KD (35.4 ± 4.8 IU/L, *P* < 0.05) and UTI patients (66.0 ± 8.0 IU/L, *P* < 0.01) than in VI patients (17.0 ± 6.2 IU/L). In the isozyme pattern analysis, KD patients had high levels of U-LDH1 and U-LDH2, while UTI patients had high levels of U-LDH3, U-LDH4, and U-LDH5. Furthermore, IVIG nonresponders of KD had significantly higher levels of total U-LDH activity (45.1 ± 4.7 IU/L, *P* < 0.05), especially U-LDH1 and U-LDH2 (*P* < 0.05), than IVIG responders (32.0 ± 2.8 IU/L). KD patients have increased levels of total U-LDH activity, especially U-LDH-1 and U-LDH2, indicating a unique pattern of U-LDH isozymes different from that in UTI patients.

## 1. Introduction

Kawasaki disease (KD) is an acute febrile illness that predominantly affects infants and children [1]. This disease is characterized by a systemic vasculitis that may have coronary artery lesions (CALs) [1, 2]. Although intravenous immunoglobulin (IVIG) is the established treatment for acute KD [2, 3], more than 10% of such patients are resistant to this therapy. KD has a diverse array of clinical manifestations and laboratory findings, and the histopathological examinations demonstrate arteritis and inflammation in multiple organs including the kidney [4, 5]. Abnormal urinary findings, such as sterile pyuria, proteinuria, and microscopic hematuria, are often seen in patients with KD during the acute phase [5, 6]. Although sterile pyuria in KD had been thought to be caused by urethritis [5], Watanabe et al. have reported that these urinary cells originated from both the urethra and the kidney as a result of mild and subclinical renal injury [7, 8].

Lactate dehydrogenase (LDH) is an enzyme found in nearly all living cells and consists of at least five isozymes (LDH1–LDH5) [9]. Urinary LDH (U-LDH) activity, especially U-LDH4 and U-LDH5, was reported to be elevated in child patients with upper urinary tract infection (UTI) [10–12]. However, there has been no report of U-LDH activity or its isozyme patterns in KD. The aim of the present study was to investigate whether or not U-LDH activity is increased in the acute phase of KD and, if so, which of the isozymes are increased. Furthermore, to investigate whether or not U-LDH levels reflect the severity of KD vasculitis, the U-LDH activity and its isozyme patterns were compared between IVIG responders and IVIG nonresponders.

## 2. Materials and Methods

### 2.1. Ethical Statement

The present study was approved by the institutional review board at the National Defense Medical College.

### 2.2. Study Subjects

We retrospectively reviewed the clinical records of 120 patients with KD, 18 patients with viral infection (VI), and 43 patients with UTI. All patients were hospitalized at the National Defense Medical College hospital between January 2002 and December 2015. All KD patients were enrolled within 6 days of the onset of illness, with day 1 defined as the first day of the fever, and all patients met the diagnostic criteria for KD established by the Diagnostic Guidelines for Kawasaki Disease (5th revision) [13]. No bacterial species were identified in the blood or urine cultures from KD patients. All KD patients were treated with oral aspirin (30 mg/kg/day), IVIG (2 g/kg/day), and intravenous ulinastatin (15000 U/kg in 3 divided doses) [14]. “IVIG nonresponder” was defined as a persistent fever lasting >24 h after the completion of IVIG or a recrudescent fever associated with KD symptoms after an afebrile period. The Kobayashi score [15], which is a common predictor for IVIG resistance in Japan, was calculated in all KD patients. Although six patients had transient dilatation of coronary arteries from the acute through the subacute phases, they had no segmental aneurysm at the convalescent phase. Urine samples were obtained from KD patients in the acute febrile phase (pre-IVIG), subacute phase (post-IVIG), and convalescent (afebrile) phase. U-LDH activity was analyzed using the Japanese Society of Clinical Chemistry (JSCC) transferable method [16]. U-LDH isozyme was analyzed using a cellulose acetate membrane electrophoresis.

The VI group included 18 children with fever (≥38°C). The type of virus in three patients was definitively diagnosed (two with adenovirus infection by a rapid diagnostic test using throat swab and one with Epstein-Barr virus infection by a serum antibody test). As 15 patients (9 with acute upper respiratory infection and 6 with bronchopneumonia) had low CRP levels (<2.0 mg/dl) and their fever went down without the administration of antibiotics, all of them were clinically diagnosed with some type of viral infection. None of the VI patients met the diagnostic criteria for KD, and no bacterial species were detected in the urine culture from them.

The UTI group included 43 children with a fever (≥38°C) before antibiotic therapy. Urinary specimens for culture were collected by catheter. The diagnosis of UTI was based on a quantitative urine culture yielding greater than 10^5^ colony-forming units (CFU) of bacteria per milliliter of urine. The UTI group included 37 children with* Escherichia coli*, 4 with* Klebsiella oxytoca*, and 2 with* Enterococcus faecalis*, detected in urine culture. Thus, all UTI patients were diagnosed as upper UTI.

### 2.3. Statistical Analysis

All of the data are presented as the mean ± standard error (SE). The statistical analyses were performed using JMP11-0 statistical software (SAS Institute, Inc., Cary, NC). Pearson's chi-square test was used for the comparison of categorical variables. We compared data among the three groups using the analysis of variance (ANOVA) tests for continuous variables and Tukey's honestly significant difference (HSD) test. We compared variables between two groups using Student's *t*-test. A *P* value < 0.05 was considered to be statistically significant.

## 3. Results

The clinical and laboratory data were compared among the KD, VI, and UTI groups (Table 1). The KD group tended to have significantly higher levels of neutrophil proportion (*P* < 0.001) and significantly lower levels of sodium (*P* < 0.01) than the VI or UTI groups. The KD group also tended to be older, have significantly higher levels of alanine aminotransferase (ALT), and have significantly lower levels of albumin (Alb) and white blood cells (WBCs) than the UTI group (*P* < 0.01).

Total U-LDH activity and its isozyme patterns were compared among the KD, VI, and UTI groups (Table 2). The UTI group had significantly higher total LDH activity (66.0 ± 8.0 IU/L, *P* < 0.01) than the KD (35.4 ± 4.8 IU/L) and VI groups (17.0 ± 6.2 IU/L), and the KD group had significantly higher total LDH activity (*P* < 0.05) than the VI group. On comparison of the U-LDH isozyme patterns among the three groups, the KD group had significantly higher levels of U-LDH1 (12.5 ± 0.9 IU/L) than the VI (5.5 ± 2.4 IU/L, *P* < 0.01) and UTI (7.4 ± 1.5 IU/L, *P* < 0.05) groups and significantly higher levels of U-LDH2 (8.1 ± 0.6 IU/L, *P* < 0.01) than the VI group (3.9 ± 1.5 IU/L). In contrast, the UTI groups had significantly higher levels of U-LDH3 (9.5 ± 1.2 IU/L), U-LDH4 (14.4 ± 2.0 IU/L), and U-LDH5 (27.4 ± 3.9 IU/L) than the KD (4.2 ± 0.7, 4.0 ± 1.2, and 6.6 ± 2.3 IU/L) and VI groups (2.0 ± 2.0, 1.6 ± 3.3, and 3.9 ± 6.4 IU/L) (*P* < 0.01). There was no correlation between the serum LDH levels and total U-LDH activity in the KD, VI, and UTI groups (data not shown).

**Figure 1:**
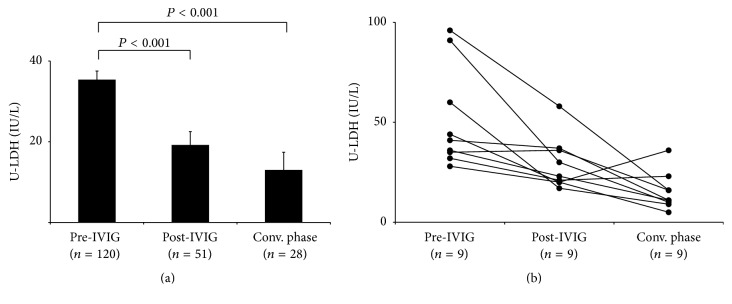
Comparison of total U-LDH activity among pre-IVIG, post-IVIG, and convalescent phases of KD (a) and the time course changes in the total U-LDH activity (b).

The total U-LDH activity was compared among the pre-IVIG, post-IVIG, and convalescent phases of KD. The mean levels of U-LDH were significantly higher in the pre-IVIG phase (35.4 ± 2.1 IU/L) than in the post-IVIG (19.2 ± 3.3 IU/L) and convalescent (13.0 ± 4.4 IU/L) phases (*P* < 0.001) (Figure 1(a)). The time course changes in the total U-LDH activity in the nine KD patients whose data could be consecutively acquired in these three phases are shown in Figure 1(b). The increased levels of total U-LDH activity in the pre-IVIG phase tended to decrease from the subacute phase throughout the convalescent phase. In the KD group (*n* = 120), 89 patients were responders to IVIG therapy, and 31 patients were nonresponders. Total U-LDH activity and its isozyme patterns during pre-IVIG phase were compared between the IVIG responders and nonresponders in the KD group (Table 3). The mean Kobayashi score in the IVIG nonresponder group (5.6 ± 0.4) was significantly higher than that in the IVIG responder group (3.5 ± 0.2) (*P* < 0.05). The IVIG nonresponder group had significantly higher total U-LDH activity (45.1 ± 4.7 IU/L, *P* < 0.05) than the IVIG responder group (32.0 ± 2.8 IU/L). Furthermore, the IVIG nonresponder group had significantly higher levels of U-LDH1 (17.4 ± 1.9 IU/L) and U-LDH2 (10.8 ± 1.1 IU/L) isozymes than the IVIG responder group (10.9 ± 1.1 IU/L, 7.1 ± 0.6 IU/L) (*P* < 0.05). Furthermore, urinary levels of N-acetyl-*β*-D-glucosaminidase (NAG, *n* = 83) and *β*2-microglobulin (*n* = 87) were measured in KD patients. There was a significant positive correlation between the total U-LDH activity and urinary NAG levels (*R* = 0.52; *P* < 0.001) but not between the total U-LDH activity and urinary *β*2-microglobulin levels (*R* = 0.22; *P* = 0.038).

## 4. Discussion

The present study revealed that total U-LDH activity increased in the acute phase of KD and decreased from the subacute phase throughout the convalescent phase. In the isozyme pattern analysis, KD patients had high levels of U-LDH1 and U-LDH2, while UTI patients had high levels of U-LDH3, U-LDH4, and U-LDH5. Furthermore, the IVIG nonresponder group of KD patients had significantly higher total U-LDH activity, especially U-LDH1 and U-LDH2 levels, than the IVIG responder group.

Total U-LDH activity has been reported to be increased in a wide spectrum of renal diseases, including an upper UTI and malignant diseases [17, 18]. The U-LDH level is reported to have no correlation with the serum LDH level [19], and the same finding was also seen in the present study. These findings suggest that U-LDH does not translocate from the bloodstream but originates from the urinary tract, including the kidney, ureter, bladder, and urethra. U-LDH is composed of five isozymes (U-LDH1–5) [9], and U-LDH1 and U-LDH2 predominate in normal, healthy children [10]. In patients with a lower UTI, such as cystitis, the predominance of U-LDH1 and U-LDH2 is maintained as it is, without significant increase in total U-LDH activity [10]. Under conditions of an upper UTI, such as pyelonephritis, the isozyme composition changes from predominance of U-LDH1 and U-LDH2 (fast-zone pattern) to that of U-LDH4 and U-LDH5 (slow-zone pattern) [10, 11]. Since leukocytes contain abundant LDH4 and LDH5, the presence of elevated numbers of leukocytes in the urine may result in a shift to the predominance of U-LDH4 and U-LDH5 [9]. Furthermore, it has been reported that infection, ischemia, or necrosis can alter the isozyme composition of renal tissue [11].

The present study is the first to find that KD patients had high levels of U-LDH activity. The predominance of U-LDH1 and U-LDH2 isozymes in the acute phase of KD is thought to be derived from the kidney and urinary tract. Amano et al. reported that urinary abnormalities such as proteinuria and pyuria in KD may be caused by inflammation in the urinary system including focal interstitial nephritis, cystitis, and prostatitis [20]. Ohta et al. demonstrated that the urinary levels of urinary NAG and *β*2-microglobulin were elevated during the acute phase of KD, suggesting the presence of an inflammatory process within the renal parenchyma, such as interstitial nephritis [21]. The present study revealed that the U-LDH activity was positively correlated with the urinary levels of NAG. Renal echographic evaluation demonstrated enlarged kidneys with increased corticomedullary differentiation [22], and follow-up technetium-99m dimercaptosuccinic acid scintigraphy single-photon-emission computed tomography revealed renal scarring in 46% patients of KD [23]. Wu et al. reported that immune-mediated vasculitis may be responsible for renal involvement in KD [24]. Therefore, the elevation of U-LDH1 and U-LDH2 isozymes may be mainly caused by renal injury due to KD vasculitis. However, because not all of KD patients have urinary abnormalities, such as proteinuria, pyuria, or increased levels of NAG, *β*2-microglobulin, and U-LDH, these renal findings do not directly lead to the diagnosis of KD.

Our results also revealed that the IVIG nonresponder group had significantly higher U-LDH activity, especially U-LDH1 and U-LDH2 isozymes, than the IVIG responder group. IVIG nonresponders are suggested to be at higher risk of developing CALs than IVIG responders [2, 3]. Fatal KD cases are reported to have findings of renal vasculitis, hypercellularity in glomeruli, and interstitial cell infiltration [5]. Recently, the new term “Kawasaki disease shock syndrome” has been proposed as a severe form of KD, and these patients are reported to be resistant to IVIG therapy and associated with greater risk of CALs [25]. Gatterre et al. reported that 10 out of 11 patients with Kawasaki disease shock syndrome developed acute kidney injury [26]. Thus, it cannot be denied that these high levels of U-LDH1 and U-LDH2 may reflect the degree of severity of this disease.

The patterns of U-LDH isozymes differed between the KD and UTI groups (Table 2). Namely, U-LDH1 and U-LDH2 dominated in KD, while U-LDH3, U-LDH4, and U-LDH5 dominated in UTI. Benseler et al. reported that 42 (33%) out of 129 patients with KD had concurrent infections at the time of diagnosis, and 4 patients had UTIs due to* E. coli* and* Klebsiella* [27]. Wu et al. revealed that 8 (10.7%) out of 75 patients with KD had bacterial pyuria in urine culture, indicating that pyuria is not always sterile in KD patients [28]. There is a case report of KD misdiagnosed as acute pyelonephritis, because fever and pyuria preceded the appearance of classical mucocutaneous signs [29]. Therefore, the measurement of U-LDH isozymes may aid in the differential diagnosis of KD and UTI.

Several limitations associated with the present study must be mentioned. First, we investigated a small number of patients in only one institution. The VI group is thought to include a wide variety of viral infections, and the number of patients was small. To determine the sensitivity and specificity of U-LDH for distinguishing KD from UTI and other infectious diseases, further studies will be needed in the future. Second, since the present study was a retrospective observation without randomization, it may have had some bias. It is possible that the patients in the present study were not representative of patients with KD in general. A prospectively designed study should be performed to minimize the survey bias. Third, since none of KD patients had CALs as sequelae in the present study, we could not investigate the association between U-LDH level and CALs. Further studies will be needed to determine whether or not U-LDH level is a predictor for CALs. Despite these limitations, however, the present results offer new information that KD patients have increased total U-LDH activity, especially U-LDH1 and U-LDH2 isozymes, unlike UTI.

In conclusion, the total U-LDH activity increased in the acute phase of KD and decreased from the subacute throughout the convalescent phases. While UTI patients had high levels of U-LDH3, U-LDH4, and U-LDH5, KD patients had high levels of U-LDH1 and U-LDH2. IVIG nonresponders with KD had significantly higher total U-LDH activity, especially U-LDH1 and U-LDH2, than IVIG responders. These results indicate that KD patients have a unique pattern of U-LDH isozymes that differ from those in UTI patients.

## Figures and Tables

**Table 1:** A comparison of the clinical data among the KD, VI, and UTI groups.

	KD (*n* = 120)	VI (*n* = 18)	UTI (*n* = 43)	*P*
Male, *n* (%)	70 (58.3)	10 (55.6)	28 (65.1)	0.689^#^
Age, month	33.4 ± 2.78^†^	41.1 ± 7.22	11.9 ± 4.63	<0.001
T-Bil, mg/dl	1.24 ± 0.12	0.76 ± 0.47	0.65 ± 0.21	0.060
AST, IU/L	150.1 ± 23.6	45.4 ± 97.1	42.3 ± 43.8	0.089
ALT, IU/L	138.0 ± 16.4^†^	28.0 ± 67.2	28.2 ± 30.3	0.006
LDH, IU/L	349.8 ± 13.6	305.9 ± 54.6	283.0 ± 24.8	0.074
TP, g/dl	6.50 ± 0.07	6.46 ± 0.20	6.49 ± 0.10	0.980
Alb, g/dl	3.77 ± 0.05^†^	4.03 ± 0.17	4.16 ± 0.08	<0.001
Sodium, mEq/L	134.5 ± 0.25^*∗∗*^	138.3 ± 2.05	137.2 ± 0.47	<0.001
CRP, mg/dl	9.05 ± 0.47	2.36 ± 1.65	6.51 ± 0.78	<0.001
WBC, ×10^3^/*μ*L	15.0 ± 0.55^†^	13.2 ± 2.18	19.6 ± 1.01	<0.001
Neutrophil, %	71.9 ± 1.40^*∗*^	42.8 ± 5.74	59.5 ± 2.57	<0.001
Hb, g/dl	11.4 ± 0.12	11.8 ± 0.42	11.4 ± 0.18	0.680
Hct, %	34.2 ± 0.27	34.5 ± 1.15	33.2 ± 0.51	0.194
Plt, ×10^4^/*μ*L	38.3 ± 2.59	43.3 ± 11.2	44.5 ± 4.80	0.521

Alb, albumin; ALT, alanine aminotransferase; AST, aspartate aminotransferase; CRP, C-reactive protein; Hb, hemoglobin; Hct, hematocrit; LDH, lactate dehydrogenase; Plt, platelet; T-Bil, total bilirubin; TP, total protein; WBC, white blood cell.

The data are presented as the mean ± SE for continuous variables and as the number of patients (%) for categorical variables.

The *P* values were obtained using an ANOVA or ^#^Pearson's chi-squared test.

^*∗*^*P* < 0.001 versus VI and UTI, ^*∗∗*^*P* < 0.01 versus VI and UTI, and ^†^*P* < 0.01 versus UTI were obtained using Tukey's HSD test.

**Table 2:** A comparison of U-LDH and its isozyme patterns among KD, VI, and UTI groups.

	KD	VI	UTI	*P*
	(*n* = 120)	(*n* = 18)	(*n* = 43)	
U-LDH, IU/L	35.4 ± 4.8^*∗*^	17.0 ± 6.2	66.0 ± 8.0^††^	0.002

U-LDH1, IU/L	12.5 ± 0.9^*∗*†^	5.5 ± 2.4	7.4 ± 1.5	0.002
U-LDH2, IU/L	8.1 ± 0.6^*∗*^	3.9 ± 1.5	7.1 ± 0.9	0.039
U-LDH3, IU/L	4.2 ± 0.7	2.0 ± 2.0	9.5 ± 1.2^††^	<0.001
U-LDH4, IU/L	4.0 ± 1.2	1.6 ± 3.3	14.4 ± 2.0^††^	<0.001
U-LDH5, IU/L	6.6 ± 2.3	3.9 ± 6.4	27.4 ± 3.9^††^	<0.001

The *P* values were obtained using an ANOVA.

^*∗*^*P* < 0.05 versus VI, ^†^*P* < 0.05 versus UTI, and ^††^*P* < 0.01 versus KD and VI were obtained using Tukey's HSD test.

**Table 3:** A comparison of U-LDH and its isozyme patterns between IVIG responders and nonresponders groups with KD.

	IVIG responders	IVIG nonresponders
	(*n* = 89)	(*n* = 31)
Kobayashi score	3.5 ± 0.2	5.6 ± 0.4^*∗*^

U-LDH, IU/L	32.0 ± 2.8	45.1 ± 4.7^*∗*^

U-LDH1, IU/L	10.9 ± 1.1	17.4 ± 1.9^*∗*^
U-LDH2, IU/L	7.1 ± 0.6	10.8 ± 1.1^*∗*^
U-LDH3, IU/L	3.9 ± 0.4	5.0 ± 0.7
U-LDH4, IU/L	3.8 ± 0.6	4.4 ± 1.0
U-LDH5, IU/L	6.4 ± 0.8	7.5 ± 1.4

^*∗*^*P* < 0.05 versus IVIG responders was obtained using Student's *t*-test.

## References

[1] Kawasaki T., Kosaki F., Okawa S., Shigematsu I., Yanagawa H. (1974). A new infantile acute febrile mucocutaneous lymph node syndrome (MLNS) prevailing in Japan. *Pediatrics*.

[2] Newburger J. W., Takahashi M., Beiser A. S. (1991). A single intravenous infusion of gamma globulin as compared with four infusions in the treatment of acute Kawasaki syndrome. *New England Journal of Medicine*.

[3] Nakamura Y., Yashiro M., Uehara R. (2012). Epidemiologic features of Kawasaki disease in Japan: results of the 2009-2010 nationwide survey. *Journal of Epidemiology*.

[4] Ogawa H. (1985). Kidney pathology in muco-cutaneous lymphnode syndrome. *Japanese Journal of Nephrology*.

[5] Burns J. C. (2001). Kawasaki disease. *Advances in Pediatrics*.

[6] Melish M. E., Hicks R. M., Larson E. J. (1976). Mucocutaneous Lymph Node Syndrome in the United States. *American Journal of Diseases of Children*.

[7] Watanabe T., Abe Y., Sato S., Uehara Y., Ikeno K., Abe T. (2007). Sterile pyuria in patients with Kawasaki disease originates from both the urethra and the kidney. *Pediatric Nephrology*.

[8] Watanabe T. (2015). Pyuria in patients with Kawasaki disease. *World Journal of Clinical Pediatrics*.

[9] Dubach U. C. (1966). On the origin of lactic dehydrogenase isoenzymes in urine. *Helvetica Medica Acta*.

[10] Carvajal H. F., Passey R. B., Berger M., Travis L. B., Lorentz W. B. (1975). Urinary lactic dehydrogenase isoenzyme 5 in the differential diagnosis of kidney and bladder infections. *Kidney International*.

[11] Devaskar U., Montgomery W. (1978). Urinary lactic dehydrogenase isoenzyme IV and V in the differential diagnosis of cystitis and pyelonephritis. *Journal of Pediatrics*.

[12] Lorentz W. B., Resnick M. I. (1979). Comparison of urinary lactic dehydrogenase with antibody-coated bacteria in the urine sediment as means of localizing the site of urinary tract infection. *Pediatrics*.

[13] Ayusawa M., Sonobe T., Uemura S. (2005). Revision of diagnostic guidelines for Kawasaki disease (the 5th revised edition). *Pediatrics International*.

[14] Kanai T., Ishiwata T., Kobayashi T. (2011). Ulinastatin, a urinary trypsin inhibitor, for the initial treatment of patients with Kawasaki disease: a retrospective study. *Circulation*.

[15] Kobayashi T., Inoue Y., Takeuchi K. (2006). Prediction of intravenous immunoglobulin unresponsiveness in patients with Kawasaki disease. *Circulation*.

[16] Japan Society of Clinical Chemistry (1990). Recommendation for measuring enzyme activity in human serum. Lactate dehydrogenase (1989-08-30). *Japanese Journal of Clinical Chemistry*.

[17] Rosalki S. B., Wilkinson J. H. (1959). Urinary lactic dehydrogenase in renal disease. *The Lancet*.

[18] Wacker W. E. C., Dorfman L. E. (1962). Urinary lactic dehydrogenase activity. I. screening method for detection of cancer of kidneys and bladder. *The Journal of the American Medical Association*.

[19] Crockson R. A. (1961). Lactic dehydrogenase in renal disease. Urinary concentrations and relative clearances. *The Lancet*.

[20] Amano S., Hazama F., Kubagawa H., Tasaka K., Haebara H., Hamashima Y. (1980). General pathology of Kawasaki disease. On the morphological alterations corresponding to the clinical manifestations. *Acta Pathologica Japonica*.

[21] Ohta K., Seno A., Shintani N. (1993). Increased levels of urinary interleukin-6 in Kawasaki disease. *European Journal of Pediatrics*.

[22] Nardi P. M., Haller J. O., Friedman A. P., Slovis T. L., Schaffer R. M. (1985). Renal manifestations of Kawasaki's disease. *Pediatric Radiology*.

[23] Wang J.-N., Chiou Y.-Y., Chiu N.-T., Chen M.-J., Lee B.-F., Wu J.-M. (2007). Renal scarring sequelae in childhood Kawasaki disease. *Pediatric Nephrology*.

[24] Wu J.-M., Chiou Y.-Y., Hung W.-P., Chiu N.-T., Chen M.-J., Wang J.-N. (2010). Urinary cytokines and renal doppler study in kawasaki disease. *Journal of Pediatrics*.

[25] Kanegaye J. T., Wilder M. S., Molkara D. (2009). Recognition of a Kawasaki disease shock syndrome. *Pediatrics*.

[26] Gatterre P., Oualha M., Dupic L. (2012). Kawasaki disease: an unexpected etiology of shock and multiple organ dysfunction syndrome. *Intensive Care Medicine*.

[27] Benseler S. M., McCrindle B. W., Silverman E. D., Tyrrell P. N., Wong J., Yeung R. S. M. (2005). Infections and Kawasaki disease: implications for coronary artery outcome. *Pediatrics*.

[28] Wu M.-C., Jan S.-L., Lin M.-C., Fu Y.-C., Lin S.-J. (2008). Is pyuria with kawasaki disease always sterile?. *Pediatric Infectious Disease Journal*.

[29] Ristoska-Bojkovska N., Stavric K., Tasic V. (2003). Kawasaki disease misdiagnosed as acute pyelonephritis. *Pediatric Nephrology*.

